# Effects of a Digital Mental Health Program on Perceived Stress in Adolescents Aged 13-17 Years: Protocol for a Randomized Controlled Trial

**DOI:** 10.2196/25545

**Published:** 2021-04-19

**Authors:** Eliane M Boucher, Haley E Ward, Julia L Stafford, Acacia C Parks

**Affiliations:** 1 Happify Health New York, NY United States

**Keywords:** digital intervention, adolescents, stress management, mental health, mobile phone

## Abstract

**Background:**

Stress is an important transdiagnostic risk factor in adolescence and predicts a host of physical and psychological problems in adolescence and adulthood. Adolescence is also a developmental stage in which people may be more sensitive or reactive to stress. Indeed, research has shown that adolescents report high levels of stress, particularly when enrolled in school. However, adolescents report engaging in few, if any, stress management techniques. Consequently, the development of effective programs to help address adolescent stress is particularly important. To date, most stress management programs for adolescents are delivered within schools, and the evidence for such programs is mixed. Furthermore, most of these programs rely on traditional stress management techniques rather than incorporating methods to address the underlying negative cognitive processes, such as rumination, that may contribute to or exacerbate the effects of perceived stress.

**Objective:**

The aim of this study is to test the short-term effects of a digital mental health program designed for adolescents aged 13-17 years on perceived stress and rumination.

**Methods:**

This is a randomized controlled trial in which adolescents between the ages of 13 and 17 years, with elevated levels of perceived stress and brooding, will be randomly assigned to complete 8 weeks of a digital mental health program (Happify for Teens) or to a corresponding wait-list control group. The study will take place over 3 months, including the 8-week intervention period and 1-month postintervention follow-up. The primary outcome, perceived stress, along with secondary and exploratory outcomes (ie, brooding, optimism, sleep disturbance, and loneliness) will be assessed via self-report at baseline, 4 weeks, 8 weeks, and 12 weeks to compare changes in these outcomes across conditions.

**Results:**

Recruitment is expected to begin in the second quarter of 2021, with a target sample size of 800 participants (400 per condition). Participants will begin the study as they are recruited and will finish in waves, with the first wave of data expected 8 weeks after recruitment begins and the final wave of data expected by the end of the third quarter of 2021.

**Conclusions:**

Although school-based stress management programs for adolescents are common, research suggests that they may be limited in their reach and more effective for school-based stress than other types of stress. This trial will be one of the first attempts to examine the potential benefits of a digital mental health program on adolescents to address stress along with negative cognitive processes such as rumination. If successful, this would help introduce a more scalable alternative to school-based programs that offers adolescents greater privacy while also providing insight into novel ways to target adolescent mental health more generally.

**Trial Registration:**

ClinicalTrials.gov NCT04567888; https://clinicaltrials.gov/ct2/show/NCT04567888

**International Registered Report Identifier (IRRID):**

PRR1-10.2196/25545

## Introduction

### Background

In the United States, approximately 16.5%, or 7.7 million, adolescents have at least one mental health disorder [1]. Worldwide, approximately 10%-20% of adolescents struggle with mental health problems [2]. Importantly, the rates of mental illness have increased among adolescents in recent years [3] and at steeper rates than among adults [4]. Given that the onset of many mental health disorders occurs in childhood and adolescence, which often continue into adulthood [5,6], addressing mental health in adolescence is a key factor for reducing the prevalence of mental health disorders in adulthood.

Despite several public health efforts to increase access to mental health services for youth, service utilization by adolescents with mental health disorders remains low [7]. Only 50% of American adolescents with mental illness seek mental health treatment [1], and only 36.2% actually receive treatment [7]. The number of adolescents receiving treatment for internalizing disorders, such as anxiety and depression, is even lower [7,8]. Paradoxically, national trends indicate that, overall, adolescent use of outpatient mental health services, including both psychotherapy and psychotropic medications, has increased over time [9]. However, this increase is primarily attributed to adolescents with less severe or no mental health impairment [9]. This is problematic given the shortage of mental health service providers for children and adolescents, particularly in rural and low-income areas [10]. The increased use of these services by adolescents in general likely places a strain on the available services for youth, and, in turn, adolescents with more serious mental health disorders may have more trouble receiving necessary treatment.

### Mental Health Prevention Programs for Adolescents

Although discussions of mental health often focus on dysfunction, more recently, there has been a call for a definition of mental health that considers more than the mere absence of mental illness [11]. For example, Keyes [12] argued for a distinction between *languishing*, or the absence of mental health, and *flourishing*, or the presence of mental health. In other words, mental health also encapsulates positive or protective features, including well-being, optimal prosocial functioning, emotion regulation, empathy, and resilience [11,13]. Consequently, addressing mental health concerns in adolescents should also include efforts to promote flourishing, as this may help to reduce the likelihood of future dysfunction [13].

However, there has been more attention on mental health treatment in youth than on prevention [2]. Given the strain on mental health services for youth from adolescents with less severe or no impairment, interventions focused more on promoting flourishing and preventing future dysfunction and impairment are important [13]. Such programs can be universal, which are directed at all adolescents and often offered in schools, or targeted, in that they focus on adolescents who are at risk of more severe impairment [2]. Both universal and targeted programs can help address the growing interest in mental health services by adolescents while reserving more intense treatment programs for youth with mental illness, for whom prevention programs are not sufficient [13]. In particular, interventions that target transdiagnostic risk factors, which predict the onset and maintenance of multiple disorders [14], are likely to have the greatest effect in terms of preventing future dysfunction [15,16].

Research suggests that these prevention programs have both concurrent and long-term benefits for mental health, particularly when they target specific risk and protective factors for adolescent mental health [2,17]. For example, universal resilience-based interventions delivered in schools reduce internalizing symptoms in adolescents aged 11-18 years [18]. In another study, students who were offered a universal, positive psychology intervention reported significant reductions in distress, anxiety, and depressive symptoms, whereas students who were not offered this intervention reported increases in all these outcomes [19]. Targeted prevention programs based on cognitive behavioral therapy (CBT) have also been shown to have short-term effects on depressive symptoms [20,21] and improve self-efficacy and academic achievement in adolescents [22].

### Stress as a Risk Factor in Adolescence

Over the past decade, researchers have become increasingly interested in adolescent stress as a transdiagnostic risk factor because of its prevalence and its association with internalizing and externalizing disorders [23-25] as well as a host of other negative consequences, including poorer physical and academic outcomes. Adolescence is also a developmental stage where individuals may be particularly sensitive to stress due to shifts in hypothalamic-pituitary-adrenal axis reactivity, leading to more intense hormonal responses to stressors, particularly in older adolescents [26].

Although animal models suggest that predictable, chronic mild stress levels in adolescence can actually increase resilience in adulthood [27], research suggests that many adolescents are coping with more severe stress levels. For example, in one study, 22% of adolescents aged 15-17 years reported moderate to severe levels of perceived stress [28]. In addition, a national survey conducted by the American Psychological Association (APA) in 2013 found that adolescents reported higher levels of stress than they perceived to be healthy and that their stress levels during the school year exceeded those of adults [29]. In addition, 31% of these adolescents reported that their stress levels had increased over the previous year, whereas 34% believed that these would increase over the following year [29]. These high stress levels may reflect a variety of stressors in adolescence. Qualitative interviews with adolescents suggest that they perceive their stress to stem from family and academic sources as well as role transitions and societal problems [30]. Quantitative research corroborates these accounts, suggesting that adolescents’ primary sources of stress are school and arguments at home [31]. In fact, adolescents report higher levels of stress during the school year than during the summer, with more than twice as many adolescents reporting extreme levels of stress during the school year than during the summer break [29].

Regardless of the source, such high levels of stress can have deleterious effects on adolescents’ psychological and physical health, including lower life satisfaction [32], poorer academic performance [33,34], cigarette smoking [35], emotional eating [36], poorer diet [37], and more frequent subjective health complaints (eg, headaches, fatigue, and sleep difficulties) [38]. Stress in adolescence has also been linked to internalizing symptoms, including depression [23,24] and anxiety [24]. Interpersonal stress, in particular, predicts the onset of the first major depressive episode among adolescents [39]. Thus, addressing stress in adolescents is critical for avoiding long-term problems. Although the frequency of stressful or negative life events predict negative outcomes [35,39], adolescents’ cognitive appraisal of these events, that is, their perceptions of stress, also predicts negative outcomes [32,36-38]. Perceived stress may be more strongly tied to negative outcomes than to life events alone [40].

However, adolescents tend not to believe that stress negatively affects their mental health, and most do not engage in regular stress management [29]. Approximately 55% of adolescents report setting aside time for stress management only “a few times a month or less,” 13% report never setting aside time for stress management, and only 5% report having seen a mental health professional about their stress [29]. On the basis of these data, the APA noted the need for opportunities to help adolescents address and cope with their stress “to break this unhealthy legacy of stress in America” [29].

Although there has been an increase in interventions targeting perceived stress in adolescence [41], they are much less common than those for anxiety and depression [42]. Furthermore, most interventions for stress are school-based, and the evidence for these interventions is mixed. In a systematic review of stress management interventions, only 58% of the reviewed studies found significant improvements in physiological indicators of stress (eg, blood pressure) or self-reported stress [43]. More recent meta-analyses of school-based programs suggest that the benefits may be limited to targeted samples (eg, with high levels of stress) and not universal programs [41,42] and may only be effective for school rather than social stress [41]. Given these findings, we need more research exploring the effectiveness of stress management programs, particularly those that could be delivered outside schools.

### Negative Cognitions and Stress

Research suggests that stress in adolescence stems, at least in part, from feelings of helplessness and negative affect [44]. Therefore, effective interventions for adolescent stress may need to target the underlying negative cognitions as well as perceived stress. However, recommended approaches to addressing stress tend to focus on stress management training, relaxation training, and problem-solving and decision-making skills training [16,41,42], which may not adequately address the related negative cognitions.

Indeed, there is evidence that improving the content of adolescents’ cognition can help mitigate the negative effects of stress. For example, interpersonal stress predicts depressive symptoms in adolescents only when coupled with negative cognitions [39] or persistent low positive affect [45], whereas self-compassion buffers against the negative effects of perceived stress on internalizing symptoms [24]. In other words, addressing both negative cognitions and stress together may be particularly important in mitigating the negative consequences of stress [39].

The cognitive processes related to these negative cognitions may also be relevant. Rumination is a pattern of thinking in which individuals repeatedly think about the causes, consequences, and symptoms of their negative affect and often occurs following stressful events [46]. Rumination can be beneficial, where the individual engages in reflective pondering, or harmful, where the individual engages in brooding or moody pondering [47]. Similar to stress, rumination is another important transdiagnostic risk factor in prevention interventions for adolescents, as it predicts a host of other problems [48,49]. In adolescents, rumination, and particularly brooding, predicts depressive symptoms [50,51], anxiety [52], executive functioning impairments in selective attention and attentional switching [53], substance use problems [54], posttraumatic stress disorder following traumatic events such as terrorist attacks [55], and less sleep [48]. Furthermore, rumination and psychological distress appear to be involved in a cyclical pattern whereby ruminative thinking leads to psychological distress, which then predicts more rumination [48].

As rumination can occur in response to stressful events, it also plays a role in the negative consequences of chronic stress. For instance, longitudinal research has shown that more frequent stressful life events predict increased rumination among both adults and adolescents and that rumination mediates the relationship between stressful life events and anxiety symptoms in adolescents [56]. Other studies have shown that adolescents who ruminate in response to stress are at a greater risk of depression and substance misuse [57]. In college students, stress-reactive rumination, but not momentary ruminative self-focus, predicted depressive symptoms, although both predicted depressive symptoms among students with higher levels of stress [58].

In addition to contributing to the negative effects of stress, rumination may increase perceived stress. In one study, rumination exacerbated the relationship between life hassles and depression, anxiety, and stress in adolescents [59]. Rumination also increases adolescents’ likelihood of experiencing interpersonal stress, which in turn predicts more internalizing symptoms [60]. Therefore, applying more traditional approaches such as cognitive restructuring, self-monitoring, acceptance strategies, and attention control training [16] to address negative cognitive processes such as rumination may be helpful in reducing stress as well.

Unfortunately, aside from studies specifically testing stress management interventions, few intervention studies have included stress as an outcome variable [42]. To the best of our knowledge, no intervention research has focused on stress and rumination together. However, research suggests that similar approaches might be effective for reducing both stress and rumination. For example, researchers posit that mindfulness helps prevent depression by promoting low levels of rumination and high levels of self-compassion [61]. Indeed, trait mindfulness mitigates the relationship between life hassles and depression, anxiety, and stress [59], and mindfulness-based interventions have been shown to reduce perceived stress and increase self-compassion in middle school students [62]. Cross-sectional research with college students also suggests that self-compassion attenuates the effects of rumination on stress [63], and increases in self-compassion following intensive meditation retreats predict improvements in perceived stress, rumination, negative affect, and depressive symptoms [64]. CBT has also been used successfully for both rumination [49,65] and perceived stress [66], although both outcomes have never been measured together. These findings suggest that there may be a common pathway for reducing both rumination and perceived stress in adolescents. Therefore, identifying interventions that can address both would be beneficial as they would target two, rather than one, important transdiagnostic risk factors in this population.

### Objectives

The aim of this study is to test the effects of a digital mental health program that draws on several approaches to address perceived stress and rumination in adolescents. Happify for Teens is a version of the Happify Health platform that has been modified for those aged between 13 and 17 years. As in the adult version, Happify for Teens consists of gamified versions of evidence-based activities adapted from CBT [67], mindfulness-based stress reduction [68], positive psychology [69-71], behavioral activation [72,73], acceptance and commitment therapy [74], and psychoeducation [75]. Thus, Happify Health programs integrate a variety of recommended approaches to address negative cognitions [16] that appear to have promising effects for reducing perceived stress as well [62,66,76,77].

Although Happify for Teens has yet to be tested empirically, research on the adult version demonstrates that Happify Health programs lead to significant improvements in mental health. Observational studies of existing Happify Health users found significant improvements in subjective well-being after 6-8 weeks of use, with greater gains among users who completed more activities [78-80]. Similarly, participants in randomized controlled trials (RCTs) who completed at least two activities per week had significant improvements in depressive symptoms, anxiety, and resilience compared with participants in a psychoeducation control group or participants who completed less than 2 Happify Health activities per week [81,82].

Notably, although published analyses did not examine the effect of Happify Health on perceived stress directly, perceived stress was included as 1 of 3 components for a resilience index, which significantly decreased in users who competed at least two activities per week for 8 weeks [81,82]; unpublished data indicate that perceived stress specifically decreased along with resilience (Parks, AC, unpublished data, December 2018). In addition, experimental research suggests that using one of the activities within the Happify Health platform, a heart rate variability biofeedback game, following a stressful event may help users manage their stress, as evidenced by lower salivary alpha amylase in participants who completed this activity relative to controls [83]. These data suggest that Happify for Teens may help adolescents manage their stress while providing the added benefit of addressing underlying negative cognitions and cognitive processes.

To test the effect of Happify for Teens on perceived stress in adolescents, we plan to recruit adolescents aged 13-17 years who report elevated levels of perceived stress and rumination. Participants will be randomly assigned to complete 8 weeks of activities via Happify for Teens or to a corresponding wait-list control group. We will then compare changes in perceived stress at 4 weeks, 8 weeks, and 1 month post intervention across the 2 groups. We will also examine changes in brooding (the maladaptive component of rumination) as a secondary outcome. Finally, as sleep disturbance [84,85], optimism [86,87], and loneliness [88,89] have been identified as other potential transdiagnostic risk factors for future mental health impairment that may begin in adolescence, we also examined changes in sleep disturbance, optimism, and loneliness as additional exploratory outcomes.

## Methods

### Participants

This study is an RCT (NCT04567888) with a target sample size of 800 participants (400 participants per condition). Power analyses indicated that 200 participants (100 per condition) would be sufficient for 80% power to detect a small effect; however, attrition rates for this study are difficult to predict. Previous RCTs using Happify Health with adult participants had response rates ranging from 56% to 72% for an 8-week intervention and posttest [81,82]. Research on other digital interventions with adolescents has reported substantial variability in completion rates. The levels of noncompletion for internet-based CBT interventions range from 33.3% to 69.6%, with low use rates overall [90], whereas for digital interventions targeting diet and physical activity, completion rates range from 37% to 100% [91]. On the basis of this variability, we opted for a conservative approach to ensure adequate power and estimated a 75.0% (600/800) attrition rate, leading to an initial target sample size of 800 adolescents to maintain adequate power.

#### Recruitment

To recruit participants, we will advertise the study to existing Happify Health users who indicate that they have adolescent children. In addition, to target parents (and adolescents) less familiar with the platform, we will also advertise the study using targeted advertising on social media. Finally, we will distribute information about the study in 2 public schools (one located in California and another in Montana) that contacted Happify Health about the program. Representatives from these schools who contacted our research staff to express interest in the product will distribute letters to parents within their schools, with information about the study and a link to the parent prescreening survey.

Regardless of the recruitment method, interested parents will be directed to complete a brief screening questionnaire via Qualtrics to verify that their child is aged between 13 and 17 years, resides in the United States, and has never used Happify Health before. After meeting the initial eligibility criteria, parents will provide consent for each eligible child to participate in the study. In addition, we plan to use a snowballing recruitment method whereby parents who complete this screening questionnaire will be encouraged to share the study with other parents, and they will be entered into a drawing for a US $50 Amazon gift card for each parent they refer to the screening questionnaire.

We will then send eligible adolescents an email invitation to complete a separate screening questionnaire via Qualtrics, consisting of questions regarding their age, country of residence, previous Happify Health use, and about the study procedures to confirm their eligibility for the study. Respondents will also complete the Perceived Stress Scale (PSS) [40] and Ruminative Response Scale (RRS)–Short Form–Brooding Subscale [47]. Adolescents will be eligible to participate if they are aged between 13 and 17 years, currently reside in the United States, have never used Happify Health, report elevated levels of stress (ie, PSS score >14) and rumination (ie, RRS–Brooding Subscale score ≥10), and indicate that they are willing and able to complete the study activities. Participants from the same household will be permitted to participate in the study but will be instructed not to discuss the study or the platform with each other.

#### Compensation

Participants will be compensated with US $20 for completing each of the 4-week, 8-week, and 1-month postintervention assessments. Participants who complete all 4 assessments (baseline and 3 other assessments) will receive a US $20 bonus. Thus, participants will receive up to US $80 as compensation for their time; participants will be compensated in the form of an electronic gift card.

### Primary Outcome Measure: PSS

The PSS [40] is a widely used measure of the extent to which respondents view their lives as unpredictable, uncontrollable, or overloaded and has been used with adolescents in other studies [92,93]. It consists of 10 items asking participants the extent to which they have felt each of the feelings and thoughts in the previous month (eg, “In the last month, how often have you felt nervous and ‘stressed’?”), and items are rated on a scale from 0 (*never*) to 4 (*very often*). Ratings are summed so that higher scores indicate greater perceived stress.

### Secondary Outcome Measure: RRS–Short Form–Brooding Subscale

The brooding subscale of the RRS [47] examines the extent to which respondents engage in moody pondering and has been validated with adolescents [94,95]. Participants indicate how often they engage in each of the 5 behaviors (eg, “Think ‘Why can’t I handle things better?’”) on a scale from 1 (*almost never*) to 4 (*almost always*). For this study, instructions were modified so that participants can indicate the extent to which they engaged in these behaviors during the previous month. Ratings are summed so that higher scores indicate that participants engaged in more brooding during that time.

### Exploratory Outcome Measures

#### Life Orientation Test–Revised

The Life Orientation Test**–**Revised is a 10-item measure of optimism [96] that has been used with adolescents [97]. Participants indicate the extent to which they agree with each statement (eg, “In uncertain times, I usually expect the best”) on a 5-point scale, ranging from 0 (*strongly disagree*) to 4 (*strongly agree*). A total of 4 items are filler items, and ratings on the remaining 6 items can be summed to obtain an overall optimism score so that higher scores indicate more optimism.

#### Patient-Reported Outcomes Measurement Information System Pediatric Sleep Disturbance Scale–Short Form 4a

This is a 4-item scale measuring the extent to which participants experienced sleep disturbances over the past 7 days (eg, “In the past 7 days, I had trouble sleeping”), appropriate for respondents aged between 8 and 17 years [98]. Each item is rated on a scale from 1 (*never*) to 5 (*always*), and ratings are summed so that higher scores indicate more sleep disturbance or poorer sleep quality.

#### Roberts UCLA Loneliness Scale-8

The Roberts UCLA Loneliness Scale-8 [99] was adapted from the original 20-item UCLA loneliness scale [100] for use with adolescents. It consists of 8 items (eg, “I lack companionship”), and participants indicate how often each of the statements are descriptive of them on a 4-point scale from *never* (0) to *often* (3). Ratings are summed so that higher scores indicate higher levels of loneliness.

### Usage Statistics

In addition to self-reported outcomes, we also plan to collect data on usage and engagement via the Happify Health platform for those assigned to the intervention condition. Specifically, we will passively collect information about the activities completed and the number of active days.

### Procedure

This study will take place over approximately 3 months, including an 8-week intervention period and a 1-month follow-up. See Table 1 for the schedule of activities. All study procedures were reviewed and approved by IntegReview, an independent institutional review board.

After obtaining parental consent, eligible participants will be contacted via email with instructions to complete an electronic assent form and the baseline assessment. This assessment will include all primary, secondary, and exploratory outcome measures. To assess data quality, we plan to include an attention check within each questionnaire in the baseline assessment and subsequent assessments. These attention checks will instruct participants to select a specific response option to determine whether participants are reading items carefully (eg, for the PSS, an attention check item could instruct the participant to select *Never*).

Once participants have completed the baseline assessment, they will be randomly assigned to either the intervention group or the wait-list control group and will receive further instructions via email. Participants who do not complete the baseline assessment will not be assigned to a condition and will be disqualified from the study. Similarly, participants who fail all attention checks within the baseline assessment will not be assigned to a condition and will be disqualified from the study.

Participants in both conditions will receive emails or text messages throughout the study to check in and keep them engaged. At 4, 8, and 12 weeks (1 month post intervention), all participants will also be prompted to complete all primary, secondary, and exploratory measures via Qualtrics. At each assessment, participants will also be asked to indicate whether they participated in any other interventions or used any digital self-help or wellness program since the last assessment date. Participants will receive reminders if they have not completed these assessments on time, and if a participant has not completed an assessment within 7 days of its scheduled date, a member of the research team will reach out via text message or email to check in.

**Table 1:** Schedule of activities for prescreen, intervention period, and follow-up assessments.

Assessments			Time								
			Prescreen		Baseline		Week 4: midpoint assessment		Week 8: postintervention assessment		Week 12: 1-month follow-up
**Primary outcome**											
	Perceived Stress Scale	✓^a^		✓		✓		✓		✓	
**Secondary outcome**											
	Ruminative Responses Scale–Short Form–Brooding Subscale	✓		✓		✓		✓		✓	
**Exploratory outcomes**											
	Life Orientation Test–Revised	—^b^		✓		✓		✓		✓	
	Patient-Reported Outcomes Measurement System Pediatric Sleep Disturbance Scale–Short Form 4a	—		✓		✓		✓		✓	
	Roberts UCLA Loneliness Scale	—		✓		✓		✓		✓	

^a^Indicates that the outcome was assessed at this time.

^b^Indicates that the outcome was not assessed at this time.

### Intervention Group: Happify Health for Teens

**Figure 1:**
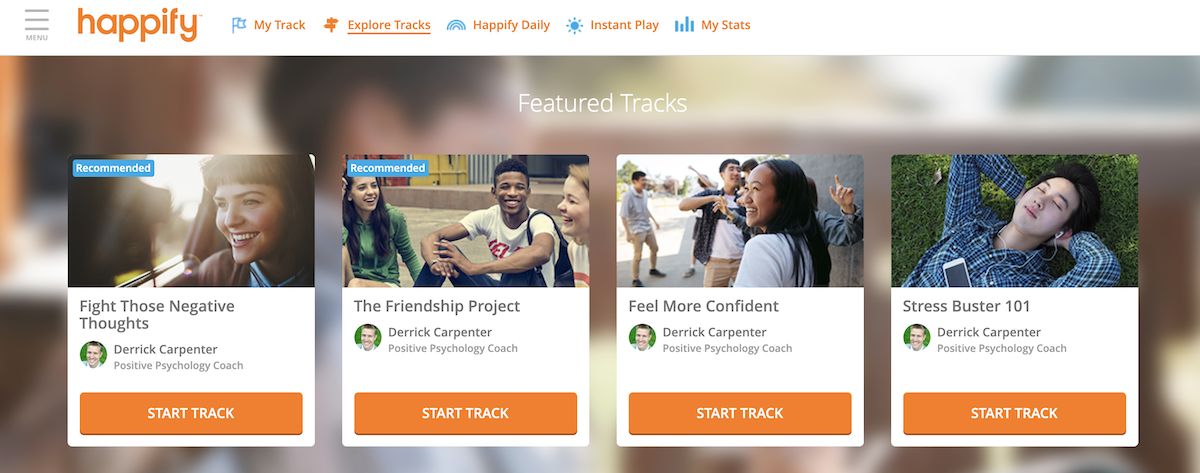
Screenshot of Happify for Teens.

Participants assigned to the active intervention will receive instructions to download Happify Health and then be directed into the Teens platform after creating an account. The Happify for Teens platform was developed using the same model as the original Happify Health platform; intervention activities are organized into 6 different skills: *savor* (mindfulness skills), *thank* (gratitude), *aspire* (optimism, goal-setting, and finding meaning or purpose), *give* (kindness, forgiveness, and prosocial behavior), *empathize* (self-compassion and perspective-taking), and *revive* (physical health). However, for the adolescent platform, these activities were modified and then reviewed by a panel of adolescents to ensure that the language and content were relevant to that population (Figure 1).

**Figure 2:**
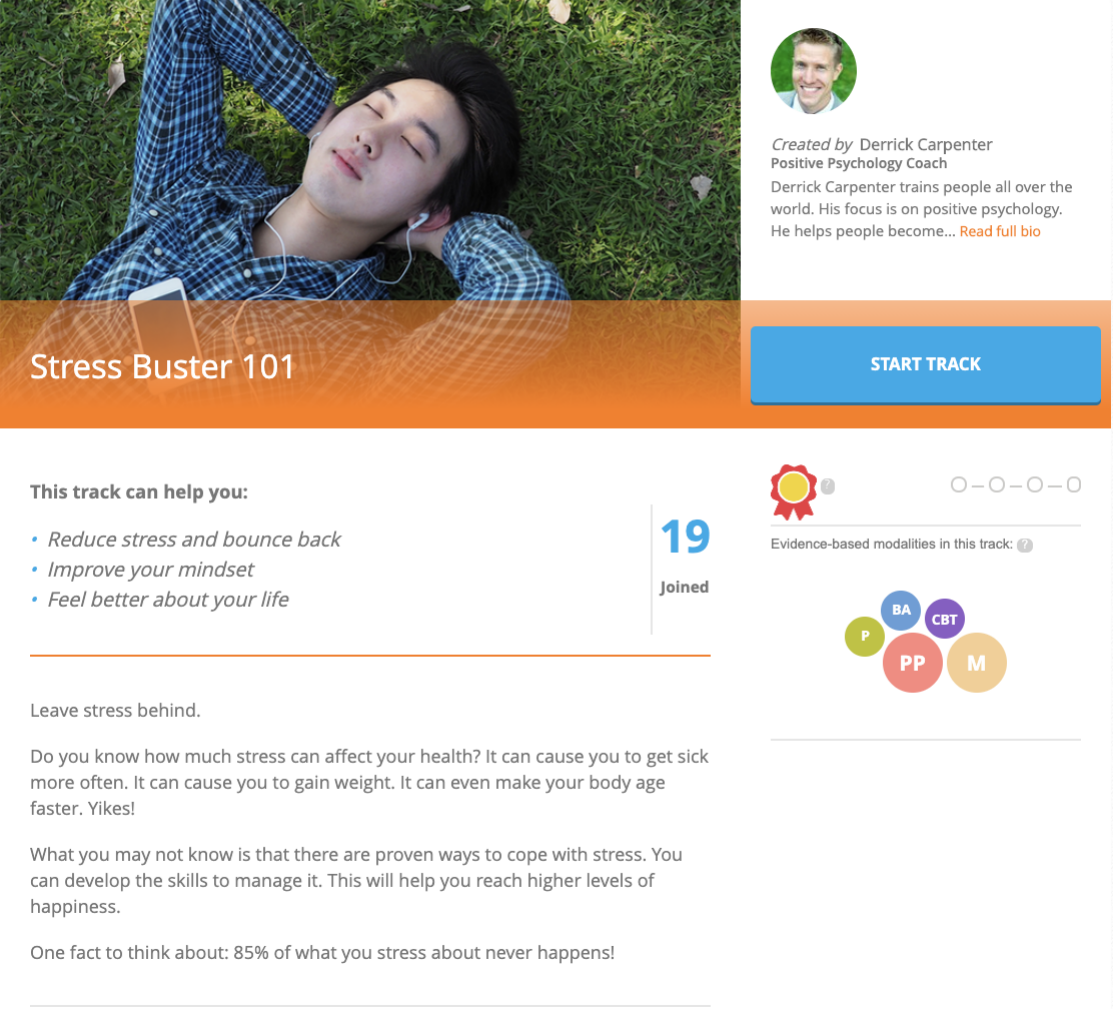
Screenshot of track description for “Stress Buster 101” track in Happify for Teens.

Activities from different categories are then organized into *tracks* focused on addressing a specific area of concern, such as increasing confidence or reducing negative thinking (see Figure 2 for a sample track description). Users can select a track that interests them, and within each track, they can choose what activities they prefer to complete, thereby offering users a more personalized experience in terms of selecting tracks as well as activities, which has been shown to improve efficacy [101,102]. Although participants will have access to all Happify for Teen tracks, we will feature a track called *Stop the Worry Cycle*. Each track consists of 4 different parts, and users must complete activities within each part to earn a silver or gold medal before they can move on to the next part. Users can also complete activities on demand through instant play and can switch tracks at any time.

Participants will not be given explicit instructions on how often they should use the platform or how many activities to complete; however, we will encourage them to engage with the platform daily. Participants will also receive push notifications on their mobile device every other day to remind them to access the platform, and they will receive weekly emails as part of the Happify Health platform to help increase engagement. If a participant has not completed any activities within the platform for 7 days, they will also receive an email or text message (based on participant preferences) from a member of the research team to check in. After the 8-week intervention period, participants in the intervention group will continue to have access to the Happify for Teens platform until the 12-week assessment but will receive no explicit instructions or no push notifications to use the program following the intervention period. We will also no longer track use to contact participants who have not used the program during this 1-month period.

### Wait-list Control Group

Active intervention or Happify Health for Teens will be compared with a wait-list control condition. Participants assigned to this condition will be instructed via email that they have been assigned to a wait-list control and that they will be given access to the Happify for Teens platform after the 12-week study period; however, they are still expected to complete the study assessments.

### Data Analysis

#### Changes in Perceived Stress and Secondary or Exploratory Outcomes

To assess whether changes in participants’ perceived stress differed across the 2 groups, we plan to conduct a 2 (group: intervention vs control)×4 (time: baseline, 4 weeks, 8 weeks, and 12 weeks) repeated-measures ANOVA (Analysis of Variance) on participants’ PSS scores, controlling for participant age, gender, and race. Similar analyses will be conducted on the secondary and exploratory outcome measures. In addition, to determine whether usage predicted changes in these outcomes, we will also conduct regression analyses among participants in the intervention condition, regressing their scores on each outcome variable on the number of activities completed within the Happify for Teens platform, while controlling for corresponding baseline scores on that outcome.

#### Rumination as a Potential Mediator

To test whether any observed changes in perceived stress can be attributed to changes in brooding, we also plan to conduct mediation analyses using Hayes PROCESS macro for SPSS [103].

#### Data Exclusion

As careless responding is problematic with web-based surveys and may artificially increase relationships among variables [104], we plan to use 2 separate a priori mechanisms to identify low-quality data: (1) failing 3 or more attention checks in an assessment and (2) completing an assessment at a rate faster than 1 second per item [104]. We will then rerun the analyses described above without these participants to determine whether any effects differ after removing low-quality data.

## Results

Recruitment is expected to begin during the second quarter of 2021 and will continue until 800 participants have been randomized to one of the two conditions. Participants will begin the 8-week intervention after being screened and consenting to participate; therefore, the first wave of data collection is expected 8 weeks after recruitment begins. We estimate that all participants will complete the intervention in the third quarter of 2021, with final completion, including the 1-month follow-up, by the end of the third quarter of 2021. Given uncertainty regarding attrition and use in an adolescent population, if fewer than 200 participants complete the postintervention assessment, we plan to conduct a second round of recruitment to increase our sample size to ensure adequate power.

## Discussion

Recent data indicate that adolescents are experiencing increasing levels of stress, particularly in school, when their stress levels exceed those of adults [29]. Given that chronic stress predicts a host of psychological, behavioral, and physical problems in adolescence [28,32-38], developing opportunities to help adolescents cope with their stress has become increasingly important [29].

Several interventions for adolescent stress have been developed; however, the evidence supporting their effectiveness is mixed [41-43]. Many of these interventions rely on traditional approaches to treating stress, including stress management training, relaxation training, and problem-solving and decision-making skills training [41,42]; however, these approaches may not adequately address negative cognitions that contribute to perceived stress [44]. For example, rumination, or maladaptive patterns of moody pondering, contributes to perceived stress [59,60] and exacerbates the negative consequences of stress [57,58]. Therefore, interventions may be particularly effective when they also incorporate approaches to addressing negative cognitions, such as cognitive restructuring and acceptance strategies [16]. To our knowledge, this is the first study to examine the effects of a stress management program on both perceived stress and rumination in adolescence and to explore whether changes in rumination mediate the effects of the program on stress.

### Evaluating the Efficacy of a Digital Prevention Intervention

Currently, most stress management programs for adolescents continue to be delivered within schools [41-43]. Although school-based prevention programs can be effective [42,43], there are numerous barriers to implementing evidence-based interventions within schools, including lack of time and resources and financial constraints [105]. Costs associated with implementing such interventions may be particularly prohibitive to schools in socially and economically disadvantaged areas [42], where students may need these interventions the most [41]. Targeted mental health interventions may have additional barriers to student participation due to fear of stigmatization [106]. Given that 95% of American adolescents own or have access to a cellular phone and 88% have daily access to a computer [107], a digital intervention addressing stress could offer a better, and arguably more cost-effective, opportunity to reach more adolescents. In addition, participation in the program would not require an entire school to buy-in; therefore, the decision to participate would be individual and private, potentially reducing concerns with stigma as well.

Although mobile mental health apps have become increasingly popular [108], mobile apps developed specifically for youth are still relatively scarce [109]. Furthermore, despite the popularity of mobile mental health apps, high quality empirical research on these apps in general is lacking [109-111]. For example, in a review of mobile mental health apps listed in the National Health Service app library, approximately 28% of mobile apps for managing depression and anxiety provided evidence to support their effectiveness claims, and only 14% of apps used validated clinical outcomes to do so [112]. Moreover, the market for these mobile apps is volatile, with one depression-focused app disappearing from the marketplace every 3 days, on average [113]. Early and robust tests of these platforms are therefore an important and necessary step to legitimize digital interventions and mobile mental health apps [114].

Empirical tests of digital interventions are particularly important with regard to platforms developed specifically for youth, as it is less clear how adolescents will respond to these interventions. Some research suggests that adolescents prefer therapy when delivered digitally rather than face-to-face [115]. However, other research suggests that adolescents report high internet and mobile app use in general but low use of mental health apps specifically [116]. Some adolescents also report feelings of being labeled and stigmatized if using a digital mental health intervention, even if no one is aware that they are using one [117]. In particular, when considering stress, adolescents tend to believe that stress has little effect on their mental health and rarely engage in stress management on their own [29]. Many studies testing digital interventions in adolescence have aimed to address poor engagement by including some degree of therapist guidance [118], phone contact [119], and physician involvement [120]; however, the effects of these strategies are inconclusive [90]. Consequently, although several studies have demonstrated the effectiveness of the general Happify Health platform with adults [78-81,92], the extent to which adolescents will engage with the digital platform, and in turn, the extent to which they will benefit from the program, is difficult to predict.

### Strengths and Limitations

Research on adolescent interventions has been characterized by low power. Although some studies of school-based stress management interventions have had large samples, a meta-analysis of 54 studies reported that 52% of the included studies had fewer than 100 participants and 72% had fewer than 200 participants [41]. Studies on digital interventions have comparatively smaller samples. For example, meta-analyses of studies on digital interventions and mobile mental health apps reported sample sizes ranging from 2 to 206 participants [90,109]. Given the mixed findings in previous research, particularly with stress management interventions [41,42], studies with larger sample sizes are needed to clarify the effectiveness of these interventions. Considering the high dropout rates with digital interventions in general [81,82,121-123], and specifically with adolescents [90,91], this study was designed to be adequately powered even with 75% attrition.

Another limitation of previous research is that the focus on school-based interventions likely results in samples that are geographically bound to schools in which these programs can be delivered, which may reduce the generalizability of those results. By comparison, testing a digital program that is accessible on adolescents’ personal computers or smartphones eliminates many barriers of school-based research, which may result in a more diverse and representative sample of adolescents.

However, our participants may differ from the general population in other ways. First, as we are advertising the study to existing Happify Health users and within specific schools that expressed an interest in the Happify Health platform, our sample may not be representative of the general adolescent population in the United States. Although this may limit the generalizability of our findings to the general population, our recruitment strategy is likely to yield a sample that is representative of Happify Health users, and consequently, our findings should be more representative of our population of interest: adolescents who will be interested in using the Happify Health platform.

In addition, with the goal of increasing retention and use, we require participants to complete the baseline assessment before qualifying for the study, and we plan to contact participants when they have not engaged with the platform or have not completed an assessment for 7 days. Consequently, our sample may include adolescents who are particularly motivated and conscientious. In addition, because we are advertising to caregivers, adolescents who participate in the study may also differ from the general population in terms of attachment style, quality of relationship with their caregiver, parenting styles, or even the caregiver’s own mental health. All these factors could affect the extent to which adolescents respond to the program [124,125].

Our sample also represents adolescents who report elevated levels of both perceived stress and brooding; therefore, it will remain unclear whether Happify for Teens would be effective as a universal stress management program (ie, for adolescents in general). Similarly, whether Happify for Teens would be effective for adolescents with either elevated levels of perceived stress or rumination, rather than elevated levels of both, will also remain unclear. Although this limits generalizability, targeted programs tend to have stronger effects than universal programs [126], and school-based stress management programs appear to be effective only with selective samples [41]. Therefore, we felt it was important to first examine whether Happify for Teens is effective as a targeted program. Arguably, given that adolescents with higher levels of perceived stress and rumination also have a higher risk of developing more severe impairment without intervention [23,24,50,52], a targeted approach also helps to test efficacy more robustly in the population that needs the program the most. Nevertheless, future research should test the effect of this program with a less selective sample of adolescents.

Previous research on digital adolescent mental health interventions has also been criticized for a lack of follow-up assessments [90,127,128]; however, some interventions, including school-based stress management interventions, may have stronger effects over time than immediately post intervention [41], emphasizing the importance of long-term follow-up. This study includes a 1-month follow-up, providing some insight into the longitudinal effects of the program, but future research should include more long-term follow-up to better ascertain whether effects diminish or strengthen over time. In addition, although reducing perceived stress and rumination should reduce participants’ likelihood of developing mental health problems, such as depression and anxiety, further research is needed to assess the long-term implications of Happify for Teens on other mental or physical health outcomes.

### Conclusions

Adolescent mental health is a growing area of concern, as many mental health problems begin in adolescence and continue into adulthood [5,6]. Prevention interventions offer a means of promoting mental health among youth before more serious dysfunction or impairment becomes an issue, thereby reducing the likelihood of mental health disorders and associated chronic disorders [2,13]. Such interventions are not only effective [2,17-22] but may also help reduce the burden on mental health services from adolescents with little to no impairment [13], who represent an increasing proportion of adolescents seeking services [9]. These interventions are most effective when they target samples with a higher risk of mental health problems [2,41] and address transdiagnostic risk factors [15,16]. Stress is an important transdiagnostic risk factor that has been studied primarily in the context of school-based stress management programs [41-43] and without considering underlying negative cognitions that might contribute to perceived stress and exacerbate the negative effects of that stress [39,44,57-60]. This trial tests a novel digital stress management program for adolescents and its effects on perceived stress and rumination. These data will, therefore, provide important information about the potential efficacy of a more scalable and cost-effective method of improving perceived stress and other associated outcomes in an adolescent population.

## Abbreviations

APA: American Psychological Association
CBT: cognitive behavioral therapy
PSS: Perceived Stress Scale
RCT: randomized controlled trial
RRS: Ruminative Response Scale
